# Influence of Psychological Distress in Patients with Hypoallergenic Total Knee Arthroplasty. Treatment Algorithm for Patients with Metal Allergy and Knee Osteoarthritis

**DOI:** 10.3390/ijerph18115997

**Published:** 2021-06-03

**Authors:** Pilar Peña, Miguel A. Ortega, Julia Buján, Basilio De la Torre

**Affiliations:** 1 Orthopedic Surgery and Traumatology Service, Virgen de la Luz Hospital, 16002 Cuenca, Spain; pilarpf1204@yahoo.es; 2 Departments of Medicine and Medical Specialities, Faculty of Medicine and Health Sciences, University of Alcalá, Alcalá de Henares, 28801 Madrid, Spain; mjulia.bujan@uah.es; 3 Ramón y Cajal Institute of Sanitary Research (IRYCIS), 28034 Madrid, Spain; bjtorre@gmail.com; 4 Department of Surgery, Medical and Social Sciences, Faculty of Medicine and Health Sciences, University of Alcalá, Alcalá de Henares, 28801 Madrid, Spain; 5 Service of Traumatology, University Hospital Ramón y Cajal, 28034 Madrid, Spain

**Keywords:** psychological distress, quality of life, total knee arthroplasty

## Abstract

The outcome in total knee arthroplasty (TKA) depends on multiples factors, among them is the psychological condition. In addition, up 15 to 30% of the patients that undergo TKA show little or no improvement after surgery, which implies the diagnosis of a painful TKA is a challenge for the orthopedic surgeon, who must rule out a possible metal allergy (MA). It is considered an exclusion diagnosis. Due to the complex relationship between psychological condition and MA, and according to the worse results in patients treated with a hypoallergenic TKA, we asked: (1). What degree of psychological distress (PD) is present in patients who have a hypoallergenic TKA, and how does it influence the results of quality of life (QoL) and functional capacity. (2). Can we develop a new algorithm for patients with a possible MA that improves the outcomes? A pragmatic clinical study was carried out that included patients who underwent hypoallergenic TKA during three consecutive years. Quality of life and functional capacity were measured with (Western Ontario McMaster Universities Osteoarthritis Index) WOMAC index, the Short Form 12 questionnaire (SF-12) questionnaire, and the The EQ-5D-5L questionnaire essentially consists of two pages: the EQ-5D descriptive system and the EQ visual analogue scale (EQ VAS) (Euro-QoL-5D L-VAS (EQ5D)), in all patients. To assess PD, a Psychological Distress Score was developed. SPSS software was performed to statistical analysis, and Student´s test for independent variables with a *p* < 0.005 as statistically significant. A total of 72 anallergic TKAs in 64 patients were treated during this period; 31.3% of these patients showed features of PD before the surgery. According with the severity of the PD, 60% were classified as severe, 10% as moderate and 30% as mild. Patients with PD had statistically significant worse results on the final WOMAC, SF-12, and EQ5D questionnaires. The final scores of the physical subscale of the SF-12 and EQ5D showed better results in patients diagnosed by psychiatrist. Up to one third of the patients with hypoallergenic TKAs have PD, and their results are clearly inferior to those patients with MA without PD. When PD was diagnosed according with Psychological Distress Score, patients should be carefully assessed in order to determine if a specialist referral is recommended. According with our results, PD should be assessed either by the PCP or by us. If the PD is confirmed, a psychiatry referral is then requested for better preoperative management and treatment. We believe that this approach would lead to better TKA outcomes.

## 1. Introduction

Total knee arthroplasty (TKA) is the definitive treatment for knee osteoarthritis (OA), reducing pain and providing a better quality of life [1,2,3,4,5,6,7]. However, 15 to 30% of the patients that undergo TKA, show little or no improvement after surgery [6,7,8]. Among other factors, this high percentage is related to the surgical technique and implant selection [1,2,9,10]. Consequently, local and systemic adverse effects related to the implant must be considered. In this aspect, metal allergy (MA), specifically to nickel (N) and chrome–cobalt (Cr-Co) stands out [10]. Metal skin hypersensitivity (MSH) is currently quite controversial in the setting of TKA. MSH is present in 25% of the painless TKAs and in up to 60% of the painful TKAs [11,12]; not being clear if detected preoperatively predisposes the patient to implant-related problems. However, despite the high incidence of MSH, 20–30% of the general population, few cases have been reported with a true hypersensitivity in the periprosthetic tissue [10,13]. On the other hand, many authors recommend implanting hypoallergenic prosthesis to avoid potential problems. Nevertheless, the diagnosis of painful TKA secondary to MA is still considered an exclusion diagnosis [14,15]. Another factor that is related to the outcomes in TKA is the psychological status of the patient. Up to one third of the patients that undergo TKA may have psychological distress (PD) [16,17], a term that encompasses a group of psychological symptoms, including anxiety, depression, and somatization, which affect the outcome of the surgery [18,19]. Due to the complex relationship of both MA and PD and according to the worse outcomes of hypoallergenic implants [17] previously published by our group, we decided to study what grade of PD is present in the patients that undergo TKA. Our primary objective was to analyse the influence that PD has on the outcomes from the hypoallergenic TKAs, and to try to develop, according to the study results, new algorithms that guide preoperative decision making and, consequently, optimize surgical results.

## 2. Patients and Methods

### 2.1. Study Design

A retrospective pragmatic clinical study was conducted to make decision in the clinic and measure the effectiveness of interventions as they are routinely used. The study was approved by the Ethical Committee and Institutional Review Board. All patients were informed on the study purposes and consent forms were obtained from the participants.

### 2.2. Patients

Inclusion criteria included patients who underwent TKA for primary knee OA between January 2013 and December 2015 at our institution. A total of 228 patients with 245 TKA were identified, being 76 of those hypoallergenic TKA (Oxinium^®^ from Smith & Nephew, Memphis, TN, USA).

**Figure 1:**
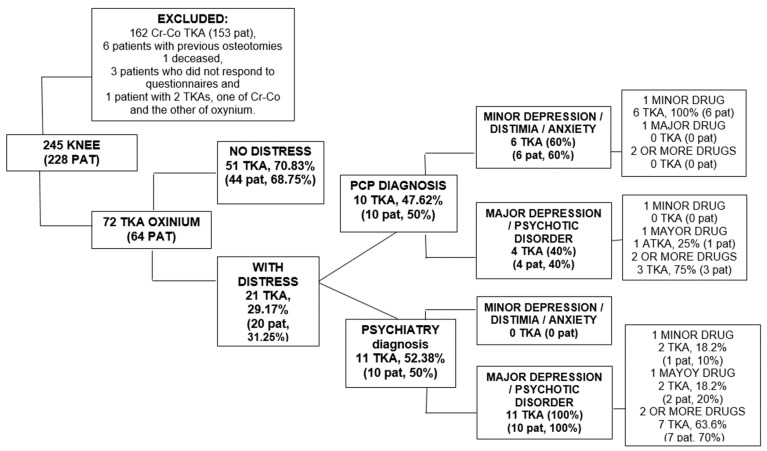
Patients flow chart with hypoallergenic TKA.

The exclusion criteria included, apart from the patients not allergic to metals with Cr-Co TKA (162 TKA in 153 patients), they were the following: patients who underwent TKA for post-traumatic OA and those with previous osteotomies around the knee [6], deceased patients [1], those who are not willing to answer the questionnaires [3] and a patient with two different implants in both knees (Cr-Co and hypoallergenic). 72 hypoallergenic RTAs in 64 patients formed the final sample of the study (Figure 1).

Preoperatively, patients were interrogated on the possibility of MSH. Those who claimed to have it, had a skin patch test (SPT) performed. If the result of the SPT was positive, an Oxinium^®^ hypoallergenic TKA was implanted at the time of surgery.

Patients were operated on by the same surgical team and with a standardized surgical technique: epidural anesthesia, prophylactic antibiotics (cefazolin 2 g IV or vancomycin 1 g 30 or 60 min before skin incision), tourniquet and medial parapatellar approach. The implant used in every case was the Genesis II (Smith & Nephew, Memphis, TN, USA), composed by a cemented Oxinium^®^ femoral component, cemented titanium tibial tray (Ti6Al4V), and a posterior-stabilized (PS) polyethylene (UHWMPE). Patellar replacement was performed in patients with Outerbridge grade IV patellar OA determined at the time of the surgery. Skin closure was performed with monofilament suture (Prolene 3/0). A drain was left in place and removed 24 h after the surgery. All patients initiated early weight-bearing 24 h after the surgery and rehabilitation was maintained for 6 weeks after hospital discharge. Patients were followed up in clinics at 4 weeks, 3, 6, 12, and 24 months.

### 2.3. Covers Variables and Data Collection

Data was collected from the medical files and included age, gender, body mass index (BMI), laterality (right/ left/ both), mental health conditions at the time of surgery, patellar prosthesis (yes/no), time from the surgery, and complications.

Time after intervention (months) is the mean time from when a patient was intervened until the telephone calls for the study were made.

Mental health conditions at the time of surgery were collected after data collection from the digital medical record (preoperative anesthesia form, form from your primary care physician (PCP) and psychiatry/psychology form, if any). Initially, those patients with PD (yes, *n* = 21 patients) or without PD (no, *n* = 51 patients) were determined. Subsequently, we made an assessment of the type of PD they suffered through the questionnaire carried out together with the Psychiatry Service.

### 2.4. Questionnaires

Quality of life and function were evaluated by three questionnaires: Western Ontario and MacMaster (WOMAC) OA Index [2,9,16,19,20,21], health questionnaire Short-Form-12 (SF-12) [19,22,23,24,25,26], and Euro-QoL-5D L-VAS (EQ5D) [27,28,29].

To assess preoperative PD, a Psychological Distress Score was developed along with the Psychiatry Department. This is a tool created ad hoc for this study. Three parameters were considered for its elaboration: past psychiatric history, type of condition, and number of medications taken (Table 1). Past psychiatric history was based on previous records of psychological/psychiatric diagnoses by their primary care physician (PCP) or psychiatrist. The type of condition was subdivided in two groups. First group included patients with diagnosis of minor depression, dysthymia, or anxiety. Second group incorporated those with diagnosis of major depression or psychotic disorder. The number of medications taken was subclassified in three categories: anxiolytic or minor antidepressant, major antidepressant, and two or more medications. The final sum of each of the three items gives a final score for mild (3 points), moderate (4–5 points), and severe (6–7 points) preoperative PD. The final PD, therefore, refers to the final result of the ad hoc questionnaire that each patient presents before being operated on for knee OA. Patients with no distress were assigned a 0 score, while a final score of 1–2 was impossible to obtain because those patients without mental health conditions had no diagnoses nor take medications. As those data were collected retrospectively, we decided to develop this score to avoid time-related biases that may had happened with the use of standardized prospective scales such as Hamilton, Goldberg, or PHQ-15 questionnaires. Data was retrieved from the medical files by one of the authors and by a nurse who phone called the patients. Demographic data and psychological distress were collected retrospectively while the questionnaires were completed after the study began. The postoperative PD assessment is included in the mental SF-12 and EQ-5D index questionnaires since they are questionnaires that we carry out once the patients have already been operated on.

### 2.5. Statistical Analysis

Statistical analysis was performed with the SPSS software (version 24, SPSS Inc., IBM, Armonk, NY, USA). The results are presented as mean ± standard deviation (SD) or absolute value and percentage. Student’s T test for independent variables was used to analyze if the PD had an influence in the outcomes from the questionnaires in the hypoallergenic TKAs. A *p* value of <0.05 was considered as statistically significant.

## 3. Results

### 3.1. Cohort

Table 2 summarizes the cohort characteristics of the 72 hypoallergenic TKA in 64 patients.

### 3.2. Psychological Distress in Patients with Hypoallergenic TKA

A total of 31.3% of the patients (29.17% of the TKAs) showed features of preoperative PD (Figure 1). Half of those patients were diagnosed by their PCP and half by their psychiatrist. Of the patients diagnosed by the PCP, 60% had minor depression, dysthymia, or anxiety and all had either a minor antidepressant or an anxiolytic medication. Of the 40% that had major depression or psychotic disorder, 75% were taking two or more medications and one patient had a major antidepressant. All patients that were diagnosed by Psychiatry of major depression or psychotic disorder were prescribed two or more medications in 70% of the cases.

Concerning the severity of the preoperative PD, 60% were classified as severe, 10% as moderate, and 30% as mild (Table 3).

### 3.3. Results of the Questionnaires

All comparisons are made with respect to pre-PD. We assess how the diagnosis of having or not having PD affects the results of the questionnaires (WOMAC, SF-12, and EQ-5D), the influence of PD diagnosed by the PCP or Psychiatry on the results of those questionnaires and, finally, how having minor or major depression contributes to the results of the pre-PD questionnaires.

The final WOMAC Total scores and the EQ5D index showed better results in patients without pre-PD (lower values). Furthermore, the physical and mental subscales of SF-12 and EQ5D VAS also showed better results in patients without PD (higher values in the questionnaires). All the results were statistically significant in comparison with the patients with hypoallergenic RTA and without previous PE versus the patients with PE (Table 4).

When comparing the results of the questionnaires with those that diagnosed pre-PD, the final questionnaires of WOMAC Total and the EQ5D Index showed higher (worse) and lower (worse) results in Physical SF-12 and the EQ5D VAS in patients diagnosed by their PCP. Likewise, the final score of the SF-12 Mental physical subscale showed a higher (better) result in those diagnosed by their PCP. All these results, except the mental subscale of the SF-12, were statistically significant (Table 5).

On the other hand, we confront the results of pre-PD both in patients with minor depression/dysthymia/anxiety and with major depression/psychotic disorder. WOMAC Total and EQ5D Index showed superior (worse) results in patients diagnosed with minor depression. The mental and physical subscales of the SF-12 and EQ5D VAS showed lower values (worse results) in those patients with minor depression. WOMAC Total, EQ5D Index, and EQ5D VAS were statistically significant compared to patients with hypoallergenic RTA, previous PD, and a diagnosis of minor depression (Table 6).

## 4. Discussion

To our knowledge, this is the study with the largest sample analyzing patients with hypoallergenic TKAs and PD. The relatively large sample size is due to the number of patients that self-report having MA. As stated previously, there is controversy regarding MA and implant failure. Despite SPT having a strong correlation with MA, no causal relationship has been established between dermal reactions and implant failure [30,31]. Thus, is not surprising that only 31.25% of the patients show histological changes in the periprosthetic tissue. Bravo et al. [32] were the first to deny a causal relationship between MA and TKA failure, suggesting that preoperative skin tests may not be necessary. It is not clear if a positive SPT represents a real MA. In fact, despite being quite sensitive, false negatives are present in MA tests [13,29,33]. Therefore, either cutaneous MA has no correlation with deep MA, either a positive SPT is not an adequate test to determine a deep MA. If, as we support, both premises are correct, SPT would be an unnecessary test in the setting of TKA. This could mean that even in the presence of a positive SPT is not mandatory to implant and hypoallergenic TKA. Based on this, our practice has changed and now we only ask for a SPT in those patients who self-report two or more symptoms of MA dermatitis: redness, skin inflammation, thickened-scaled skin, erythema, or local pruritus. If they do not report these symptoms, we do not perform an SPD, because just a self-reported intolerance to imitation jewelry is not enough to diagnose skin MA [34]. In this study, all patients who underwent hypoallergenic TKA underwent patch testing and gave a positive result for allergy to metals despite the fact that there are controversies in the literature with whether to perform them or not.

As the relationship between MA and PD has been reported in the past, we studied how this psychological disorder affects TKA’s outcomes [17]. Up to one third of the patients with hypoallergenic TKAs have pre-PD, and their results are clearly inferior to those patients with MA without pre-PD. This fact may be related to the association between stress and depressed immune response, both humoral and cellular, being possible that patients diagnosed with PD present a higher incidence of MA [35,36].

**Figure 2:**
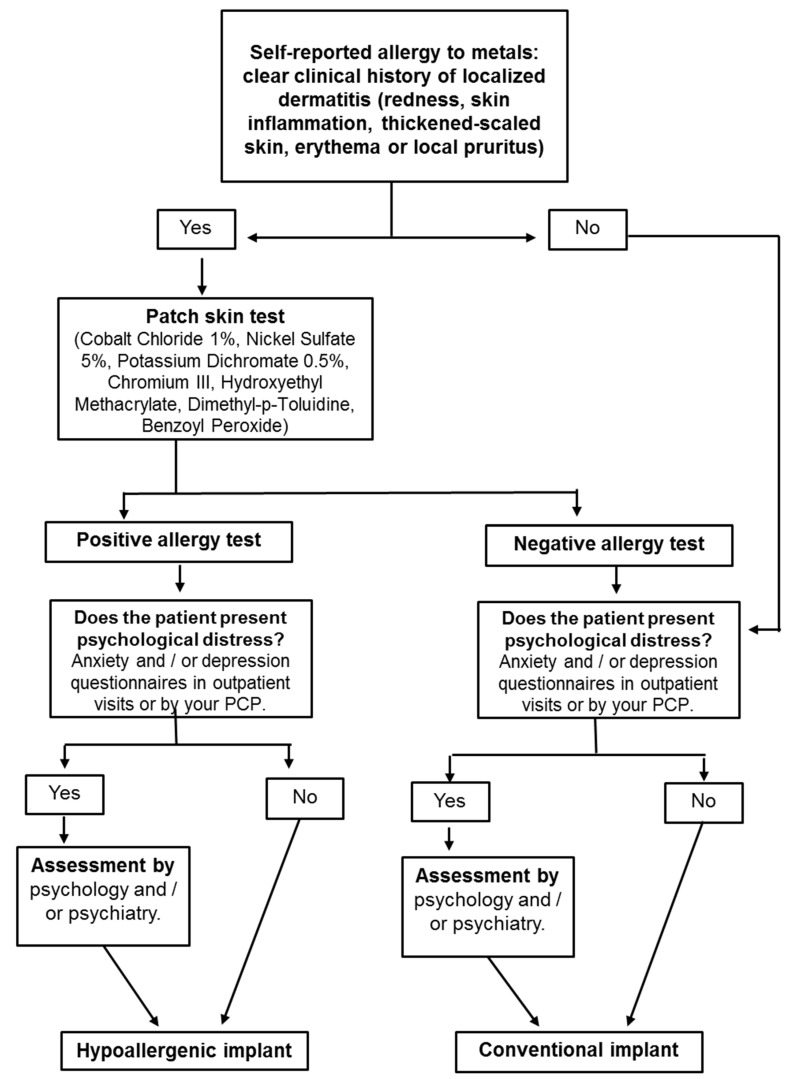
Algorithm for the diagnosis and treatment of metal allergy in a patient with knee osteoarthritis.

On the other hand, when pre-PD was diagnosed by the PCP, the WOMAC Total, SF-12 Physical, EQ5D Index, and VAS scores were significantly worse, while the SF-12 Mental was slightly better in patients previously diagnosed by their PCP, although not statistically significant between both groups. In addition, patients diagnosed with minor depression, dysthymia, or anxiety treated by their PCP had lower scores on all questionnaires than patients with major depression/psychotic disorder treated by a psychiatrist. These results may indicate that the PCP may incur underdiagnosis, misdiagnosis, or inappropriate treatment. Therefore, when a patient with MA is identified by TKA, pre-PD should be carefully evaluated to determine if referral to a specialist is recommended, since if the treatment is prescribed by a psychiatrist, better functional results could be expected (Figure 2).

The outcomes may be also influenced by the patella in those patients who had patellar resurfacing. Due to the small number of patients that had the patella prosthesis implanted (18%), we do not believe that this may had influenced the results, which is also supported by previous studies [37,38].

The expected increase in the demand of TKA in the years to come require further research on its possible failure causes. The uncertainty that surrounds MSH and its potential effects on TKA outcomes require a thorough analysis. Based on the results of our study, we recommend considering certain recommendations [34] in the patients with self-reported MA. We propose that in the setting of confirmed signs of dermatitis (redness, skin inflammation, thickened-scaled skin, erythema, or local pruritus) a SPT is performed. If these symptoms are not present we do not indicate the SPT but instead we do assess PD, due to their high association rate. In our opinion, PD should be assessed either by the PCP either by us. If the pre-PD is confirmed, a psychiatry referral is then requested for a better preoperative management and treatment. We believe that this approach would lead to better TKA outcomes.

## 5. Conclusions

In conclusion, having pre-PD means less satisfaction with the final result of hypoallergic TKA surgery in patients with MA. We highlight the importance of adequately evaluating and addressing PD in patients with self-reported MA, highlighting the fundamental role of the psychologist/psychiatrist before TKA, according to the development of our algorithm. To build a more accurate picture of the problem, further research on the topic must understand all the known factors associated with patient satisfaction.

## Figures and Tables

**Table 1:** Psychological distress score.

Psychological Distress Psychiatric History
0: no diagnosis of psychiatric pathology
1: diagnosis by family doctor
2: diagnosis by the Psychiatry Service
**Type Psychiatric Pathology**
0: no pathology
1: minor depression or dysthymia or anxiety
2: major depression or psychotic disorder
**No. and type of drugs administered to the patient**
0: no medication
1: take a minor drug
2: take a major drug
3: take two or more drugs
**Final score**
1: 3 points, mild psychiatric pathology
2: 4–5 points, moderate psychiatric pathology
3: 6–7 points, severe psychiatric pathology

**Table 2:** Descriptive characteristics of the study sample. The results are presented as the mean ± standard deviation (SD) or total value and percentage. Body Mass Index (BMI), Western Ontario McMaster Universities Osteoarthritis Index (WOMAC), Short Form 12 questionnaire (SF-12 questionnaire), The EQ-5D-5L questionnaire essentially consists of 2 pages: the EQ-5D descriptive system (Euro-Qol-5D) and Visual Analog Scale (VAS).

	TKA Hypoallergenic *n* = 72
Average age (years)	72.30 ± 6.03
Women, *n* (%)	70 (92.1)
BMI (kg/m^2^)	32.12 ± 5.08
**Weight, *n* (%)**	
Normo-weight	3 (3.9)
Overweight	24 (31.6)
Obesity	49 (64.5)
Time after intervention (months)	27.49 ±8.666
Patellar component, *n* (%)	11 (18)
**Laterality**	
Right	36 (50)
Left	23 (38.9)
Both	8 (11.1)
Psychiatric history, *n* (%)	21 (29.17)
Associated complications, *n* (%)	12 (15.8)
**WOMAC**	
Total	28.37 ± 20.66
Pain	5.27 ± 5.29
Rigidity	1.41 ± 2.10
Difficulty	22.34 ± 14.62
**SF-12**	
Physical	27.28 ± 10.58
Mental	44.86 ± 11.37
**EuroQoL-5D**	
Index	0.65 ± 0.32
VAS	53.86 ± 34.94

**Table 3:** Descriptive characteristics of the final preoperative Psychological Distress Score of hypoallergenic Oxinium TKA.

Psychological Distress Score				
Psychological Distress	3 Points, Mild	4–5 Points, Moderate	6–7 Points, Severe	Total
*N* patients (knee) (% pat)	6 (6) [30]	2 (3) [10]	12 (12) [60]	20 (21) [100]

**Table 4:** Student’s *t* results for independent samples of preoperative psychological distress in patients treated with hypoallergenic implants with respect to the results of the questionnaires (WOMAC, SF-12 and Euro-Qol-5D). PD (psychological distress: YES n = 51 TKA, 44 patients; NO n = 21 TKA, 20 patients). The results are presented as the mean ± standard deviation (SD).

	PD	TKA Hypoallergenic		
		Mean	SD	*p*
WOMAC Total	Yes	41.76	23.212	0.000
	No	23.25	17.229	
SF Physical	Yes	25.269	10.048	0.015
	No	32.804	10.058	
SF Mental	Yes	37.568	12.665	0.017
	No	42.527	9.819	
EQ Index	Yes	0.84	0.25	0.019
	No	0.71	0.28	
EQ VAS	Yes	35.14	33.600	0.013
	No	61.00	33.004	

**Table 5:** Student’s *t* results for independent samples of preoperative hypoallergenic psychological distress and TKA and diagnosis of distress by PCP or psychiatry with respect to the results of the questionnaires (WOMAC, SF-12 and Euro-Qol-5D).

	Diagnosis PD	PD and TKA Hypoallergenic		
		Mean	SD	*p*
WOMAC Total	PCP	40.03	20.72	0.046
	Psychiatry	30.24	15.275	
SF Physical	PCP	27.875	9.781	0.039
	Psychiatry	32.432	10.984	
SF Mental	PCP	40.937	10.735	0.057
	Psychiatry	38.652	9.54	
EQ Index	PCP	0.89	0.23	0.028
	Psychiatry	0.81	0.25	
EQ VAS	PCP	36.04	30.93	0.05
	Psychiatry	40.32	29.07	

**Table 6:** Student *t* results for independent samples of preoperative hypoallergenic psychological distress and TKA and diagnosis of minor depression/dysthymia/anxiety or major depression/psychotic disorder with respect to the results of the questionnaires (WOMAC, SF-12, and Euro- Qol-5D).

	Type of DS Diagnosis	PD and TKA Hypoallergenic		
		Mean	SD	*p*
WOMAC Total	Minor depression	41.32	19.793	0.039
	Major depression	31.26	16.865	
SF Physical	Minor depression	26.114	9.546	0.056
	Major depression	29.984	10.433	
SF Mental	Minor depression	36.67	9.98	0.057
	Major depression	40.154	9.17	
EQ Index	Minor depression	0.88	0.26	0.04
	Major depression	0.83	0.23	
EQ VAS	Minor depression	35.64	28.76	0.048
	Major depression	41.23	28.47	

## Data Availability

The datasets generated for this study are available on request to the corresponding author.

## Abbreviations

TKA	total knee arthroplasty
OA	osteoarthritis
MA	metal allergy
N	nickel
Cr-Co	chromium–cobalt
MSH	Metal skin hypersensitivity
PD	psychological distress
Pre-PD	preoperative Psychological Distress
BMI	body mass index
SPT	skin patch test
PS TKA	posterior-stabilized
WOMAC	Western Ontario McMaster Universities Osteoarthritis Index
SF-12	questionnaire Short Form 12 questionnaire
Euro-Qol-5D L-VAS	The EQ-5D-5L questionnaire essentially consists of two pages: the EQ-5D descriptive system and the EQ visual analogue scale (EQ VAS).
PCP	primary care physician
PHQ-15	questionnaires Patient Health Questionnaire Spanish version
SD	standard deviation
